# Extensive Capsule Locus Variation and Large-Scale Genomic Recombination within the *Klebsiella pneumoniae* Clonal Group 258

**DOI:** 10.1093/gbe/evv062

**Published:** 2015-04-10

**Authors:** Kelly L. Wyres, Claire Gorrie, David J. Edwards, Heiman F.L. Wertheim, Li Yang Hsu, Nguyen Van Kinh, Ruth Zadoks, Stephen Baker, Kathryn E. Holt

**Affiliations:** ^1^IBM Research—Australia, Carlton, Victoria, Australia; ^2^Department of Biochemistry and Molecular Biology, Bio21 Institute, University of Melbourne, Parkville, Victoria, Australia; ^3^Wellcome Trust Major Overseas Programme, Clinical Research Unit, Oxford University Hanoi, Vietnam; ^4^Nuffield Department of Clinical Medicine, University of Oxford, United Kingdom; ^5^National Hospital for Tropical Diseases, Hanoi, Vietnam; ^6^Institute of Biodiversity, Animal Health and Comparative Medicine, College of Medical, Veterinary and Life Sciences, University of Glasgow, United Kingdom; ^7^Moredun Research Institute, Pentlands Science Park, Penicuik, Midlothian, United Kingdom; ^8^Wellcome Trust Major Overseas Programme, Oxford University Clinical Research Unit, Ho Chi Minh City, Vietnam; ^9^Centre for Tropical Medicine, Nuffield Department of Clinical Medicine, Oxford University, United Kingdom; ^10^The London School of Hygiene and Tropical Medicine, London, United Kingdom

**Keywords:** *Klebsiella*, ST258, carbapenemase, *cps*, genome, evolution

## Abstract

*Klebsiella pneumoniae* clonal group (CG) 258, comprising sequence types (STs) 258, 11, and closely related variants, is associated with dissemination of the *K. pneumoniae* carbapenemase (KPC). Hospital outbreaks of KPC CG258 infections have been observed globally and are very difficult to treat. As a consequence, there is renewed interest in alternative infection control measures such as vaccines and phage or depolymerase treatments targeting the *K. pneumoniae* polysaccharide capsule. To date, 78 immunologically distinct capsule variants have been described in *K. pneumoniae.* Previous investigations of ST258 and a small number of closely related strains suggested that capsular variation was limited within this clone; only two distinct ST258 capsule polysaccharide synthesis (*cps*) loci have been identified, both acquired through large-scale recombination events (>50 kb). In contrast to previous studies, we report a comparative genomic analysis of the broader *K. pneumoniae* CG258 (*n* = 39). We identified 11 different *cps* loci within CG258, indicating that capsular switching is actually common within the complex. We observed several insertion sequences (IS) within the *cps* loci, and show further intraclone diversification of two *cps* loci through IS activity. Our data also indicate that several large-scale recombination events have shaped the genomes of CG258, and that definition of the complex should be broadened to include ST395 (also reported to harbor KPC). As only the second report of extensive intraclonal *cps* variation among Gram-negative bacterial species, our findings alter our understanding of the evolution of these organisms and have key implications for the design of control measures targeting *K. pneumoniae* capsules.

## Introduction

*Klebsiella pneumoniae* has emerged as a common cause of multidrug-resistant healthcare-associated infections. In particular, isolates of sequence type (ST) 258 and closely related variants, such as ST11 (as defined by multilocus sequence typing [MLST]; Diancourt et al. 2005), are distributed across continents (Kitchel et al. 2009; Ko et al. 2010; Gomez et al. 2011; Li et al. 2012; Richter et al. 2012; Chiu et al. 2013), cause nosocomial outbreaks (Li et al. 2012; Richter et al. 2012; Snitkin et al. 2012), and are associated with the dissemination of carbapenem resistance encoded by the *K. pneumoniae* carbapenemase (KPC) gene (Kitchel et al. 2009; Seki et al. 2011; Richter et al. 2012; Yang et al. 2013). KPC carrying *K. pneumoniae* and other carbapenem-resistant *Enterobacteriaceae* are difficult to treat (Munoz-Price et al. 2013), and have recently been recognized as an urgent public health threat by the World Health Organization (2014), the United States Centers for Disease Control and Prevention (US Department of Health and Human Services and Centers for Disease Control and Prevention 2013), and several other government bodies.

Given the limited options for therapeutic treatment of KPC and *K. pneumoniae* infections (Munoz-Price et al. 2013), there has been a resurgence of interest in *K. pneumoniae* vaccines as an alternative method of infection control, including polysaccharide vaccines targeting the capsule of outbreak strains (Ahmad et al. 2012). In addition, therapeutics based on capsule-targeting phage, or their capsular depolymerase enzymes, have also been proposed for control of *K. pneumoniae* (Lin et al. 2014). The polysaccharide capsule is among the most important *K. pneumoniae* virulence determinants, providing protection from phagocytosis, resistance to complement-mediated killing, and suppression of human beta-defensin expression (Allen et al. 1987; Kabha et al. 1995; Clements et al. 2008; Lee et al. 2014; Moranta et al. 2010). Counter-current immunoelectrophoresis techniques have distinguished 78 capsular serotypes (K types) (Brisse et al. 2004; Pan et al. 2008); however, this form of serotyping is technically challenging and rarely performed (Podschun and Ullmann 1998). The genes required for capsule biosynthesis are located at the capsule polysaccharide synthesis (*cps*) locus, which shows similarity to those of *Escherichia coli* group 1 *cps* loci (Rahn et al. 1999). Complete DNA sequences have been reported for only a minority of *K. pneumoniae cps* loci (Shu et al. 2009; Ramos et al. 2012; D’Andrea et al. 2014; Deleo et al. 2014); however, several methods of capsular typing based on genetic variation at the *cps* locus have been proposed. These include C typing, a restriction enzyme-based method that distinguishes 96 genetically distinct forms of the *cps* locus (Brisse et al. 2004), and nucleotide sequencing of the conserved genes *wzi* (encoding Wzi, which anchors capsular polysaccharide to the cell surface) (Brisse et al. 2013) or *wzc* (encoding Wzc, a tyrosine autokinase which polymerizes capsular polysaccharides) (Pan et al. 2013).

Recent reports have described the capsular diversity and molecular evolutionary history of ST258 (Chen et al. 2014; D’Andrea et al. 2014; Deleo et al. 2014; Diago-Navarro et al. 2014; Wright et al. 2014). These studies showed that ST258 descended from an ST11-like ancestor, which acquired a 1.1 Mb genomic region from an otherwise distantly related ST442-like *K. pneumoniae* via recombination (Chen et al. 2014; D’Andrea et al. 2014; Wright et al. 2014). The imported genomic region included a *cps* locus (*cps*_BO-4_), distinct from that present in the ST11 reference genome HS11286 (*cps*_HS11286_) (D’Andrea et al. 2014), and a different allele of the *tonB* MLST locus (Chen et al. 2014). Compared with its ST11 ancestor, the resulting ST11-ST442 hybrid showed a change in both capsular type and ST, and has been named ST258 clade II/ST258-2 (Chen et al. 2014; Deleo et al. 2014) or ST258b (Wright et al. 2014). Subsequently, a recombination event of approximately 50 kb arose in ST258 in which a third *cps* locus (*cps*_207-2_) was acquired by ST258-2 from an ST42-like donor, forming a new sublineage of ST258 named ST258 clade I/ST258-1 (Chen et al. 2014) or ST258a (Wright et al. 2014). These studies only included genomes of ST258 isolates and a small number of closely related variants (only three ST11) from a limited geographic distribution (mostly North America and Italy) (Chen et al. 2014; D’Andrea et al. 2014; Deleo et al. 2014; Wright et al. 2014). Overall, just three distinct *cps* loci have been characterized in clonal group (CG) 258 and two others have been indicated but not described (Chen et al. 2014).

Here, we performed a genomic investigation of 39 members of the wider *K. pneumoniae* ST258/11 CG258, which were identified within a diverse collection of 230 *K. pneumoniae* genomes as well as publicly available data. Our analysis incorporates more distantly related genomes than those included in previous reports, from nine countries across four continents, including a total of nine ST11, which is the presumed ancestor of ST258. Our analysis identifies numerous large-scale recombination events within CG258; identifies additional STs not previously recognized as part of this clonal complex; and provides independent confirmation of the recombination events involving ST42 and ST442 in the derivation of ST258. Most importantly, we identified 11 distinct *cps* loci within CG258, and used these data to explore the dynamics of capsule switching and genomic variation within the clonal complex.

## Materials and Methods

### Genome Sequence Data analyzed in This Study

Genomic data representing 39 *K. pneumoniae* CG258 representatives were included in this study (table 1). Genome assemblies for 30 isolates were retrieved from GenBank. Sequence reads for one isolate (KpMDU1) were generated on the Ion Torrent platform. A total of 76-bp paired-end (PE) sequence reads for 8 isolates were generated on the Illumina Genome Analyzer GAII platform as part of a global diversity study of *K. pneumoniae.* Accessions for all CG258 sequence data are given in tables 1 and 2.

**Table 1:** CG258 *Klebsiella pneumoniae* Isolates Included in This Study

Isolate	ST	*cps* Locus	K-Type^a^	*wzi* Allele	KPC Allele	Year	Country	Isolate Source^b^	Accession
K242An	395	A	47/NT	105	—	2005	Vietnam	Carriage	ERR025564
1191100241	395	A	47/NT	105	—	2011	Netherlands	Clinical	AFXH00000000.1
KpOXF1	437	B	UNK	109	—	2008–2011	UK	Clinical	ERR276930
KpMDU1	258-1	C	NT	29	2	2012	Australia	Clinical	AMWO00000000.1
VA360	258-1	Ci	NT	29	2	2007	USA	Clinical	ANGI00000000.2
ST258-490	258-2	D	UNK	154	3	2006	Israel	Clinical	ALIS00000000.1
ATCC BAA-1705	258-2	D	UNK	154	2	2007	Unknown	Urine	AOGQ00000000.1
NIH outbreak (*n* = 20)	258-2	D	UNK	154	3	2011	USA	Clinical	AJZU00000000.1– AKAN00000000.1
ST258-K26BO	258-2	D	UNK	154	3	Unknown	Italy	Unknown	CANR00000000.1
ST258-K28BO	258-2	D	UNK	154	3	Unknown	Italy	Unknown	CANS00000000.1
ST512-K30BO	512	D	UNK	154	3	Unknown	Italy	Clinical	CAJM00000000.2
HS11286	11	E	47	74	2	2011	China	Clinical	NC_016845.1
DM23092/04	11	F	14/64/NT	64	—	2004	Singapore	Clinical	ERS011902 (Koh et al. 2013)
DR5092/05	11	G	38	96	—	2005	Singapore	Clinical	ERS011906
ATCC BAA-2146	11	H	UNK	174	—	2010	USA/India^c^	Urine	AOCV00000000.1
NCSR101	11	I	UNK	73	—	2007	Vietnam	Clinical	ERS011807
09-370B	11	J	UNK	88	2	2009	Vietnam	Clinical	ERS012021
DU10252/04	11	K	UNK	75	—	2004	Singapore	Clinical	ERS011907
DU4033/04	11	K	UNK	75	—	2004	Singapore	Clinical	ERS011904
DU38032/05	11	Ki	UNK	75	—	2005	Singapore	Clinical	ERS011911

Note.—The National Institutes of Health (NIH) outbreak genomes (*n* = 20) are represented by a single row in the table.

NT, nontypeable; UNK, unknown serotype; – indicates no KPC allele detected in this genome.

^a^As indicated by *wzi* serotype associations reported in Brisse et al. (2013).

^b^Clinical, isolate from human infection; carriage, isolate from human anal swab; urine, isolate from human urine sample, infection status unknown.

^c^Strain isolated in the United States from a patient who had received recent medical care in India (Centers for Disease Control and Prevention 2010).

**Table 2:** Genomes Included in Additional Recombination Analyses

Isolate	ST/Sublineage	Other Designations/*cps*	*wzi* Allele^e^	Year	Country	Accession (Reference)
NJST258_1	ST258-2^a^	ST258b^b^, *cpsBO-4*^c^, *cps*-D^d^	154	2010	USA	Deleo et al. (2014)
VA360	ST258-1^a^	ST258a^b^, *cps*207-2^c^, *cps*-Ci^d^	29	2007	USA	Xie et al. (2013)
HS11286	ST11	*cps*HS11286^c^, *cps*-E^d^	74	2011	China	Liu et al. (2012)
QMP Z4-702	ST442	*cps*-D^d^	154	2006	USA	ERS012008
DB44834/96	ST42	*cps*-C^d^	29	1996	Singapore	ERS011903

^a^As in Chen et al. (2014) and Deleo et al. (2014).

^b^As in Wright et al. (2014).

^c^As in D’Andrea et al. (2014).

^d^As defined in this study.

^e^As in Brisse et al. (2013).

For genome-wide single nucleotide polymorphism (SNP) screening of potential CG258 members or recombination donors, two public read sets were also analyzed (ERP000165, ERP002642).

### Genome-Wide Phylogenetic Analysis of *Klebsiella pneumoniae*

Sequence reads or, for public genomes, 100-bp PE reads simulated from assembled sequences using SAMTools wgsim (Black et al. 2010) with 0% error rate were mapped against the HS11286 reference chromosome (accession: NC_016845.1) using Bowtie 2 (Langmead and Salzberg 2012). SNPs were identified using SAMTools (Li et al. 2009) as previously described (Holt et al. 2012). Briefly, positions in which an unambiguous SNP call was made in any isolate with Phred quality ≥30 and read depth ≥5 were identified, and consensus alleles (unambiguous homozygous base calls with Phred ≥20) were extracted from all isolates and concatenated to generate an SNP alignment. For the global phylogeny, a concatenated alignment of all 272,365 core genome SNPs was generated (defined as genome positions conserved in ≥99% of the genomes). A maximum likelihood (ML) phylogeny was generated using RAxML (Stamatakis 2006) and a general time reversible substitution model with gamma model of rate heterogeneity. The tree representing the highest ML score among 5 runs, each of 100 bootstrap replicates, is shown in supplementary figure S1, Supplementary Material online.

### Phylogeny Construction and Recombination Detection within CG258

**Fig. 1.—:**
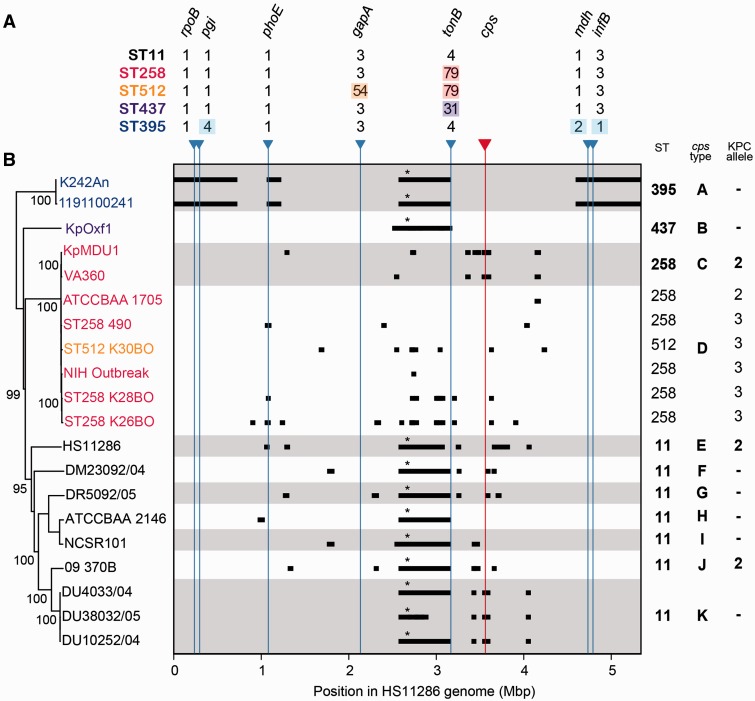
CG258 MLST profiles, genome-wide phylogeny, and putative recombinant genomic regions. Both panels (*A* and *B*) share the *x*-axis, which indicates coordinates of the HS11286 genome (ST11) that was used as the reference for read mapping and SNP calling. Blue arrows indicate the chromosomal positions of MLST loci along this axis; red arrow indicates the position of the *cps* locus. MLST alleles for the five STs are shown in (*A*); those differing from ST11 alleles are highlighted. Nucleotide polymorphisms resulting from recombinant genomic imports identified by BRATNextGen are shown as black blocks in (*B*); these were excluded from phylogenetic analysis (tree in (*B*), bootstrap support values ≥ 90% are shown (%), strain names colored as per STs in (*A*) and as marked on the right). A high resolution view of the ST258/512 subclade is shown in supplementary figure S3, Supplementary Material online. Asterisk indicates recombinant imports identified in whole or part in all non-ST258/512 representatives, which ChromoPainter analysis indicated was actually an import into ST258 (supplementary fig. S2, Supplementary Material online). Note that the reference genome is circular and thus what appears here as independent imports at the ends of the K242An and 1191100241 genomes are in fact a single import spanning the origin. Gray and white background shading indicates changes in capsular locus type; types are labeled A–K on the right, genetic structures for these are given in figure 2. STs and KPC alleles are also indicated on the right. Where isolates sharing a *cps* locus also shared an ST or KPC allele, the ST and/or KPC allele are listed once only for the entire group. Note that the NIH outbreak genomes (*n* = 20) are all highly similar and represented as a single leaf node for simplicity.

For the CG258 analysis, sequence reads or simulated reads (as above) were mapped against the HS11286 reference chromosome using Burrows-Wheeler Aligner (BWA) (Li and Durbin 2009) and SNPs were identified as above. A pseudo-whole genome alignment was generated by replacing reference bases with SNP alleles identified in each genome. Putative recombinant genome regions were identified from this whole genome alignment using BRATNextGen (Marttinen et al. 2012), with 100 × 20 iterations and a reporting threshold of *P* < 0.05. An ML phylogeny was generated using RAxML as above, using an alignment of SNPs at those sites that were conserved in ≥98% of CG258 genomes but not identified by BRATNextGen as affected by recombination (total 5,476 sites). The tree representing the highest ML score among 10 runs, each of 1,000 bootstrap replicates, is shown in figure 1. The tree was rooted using the NTUH-2044 ST23 reference genome (accession: NC_012731.1) as an outgroup.

### Investigation of Large-Scale Recombination Events Affecting ST258

Variable genome positions were identified by read mapping to the ST258 reference, NJST258_1 (accession: CP006923.1), and variant calling as described above. The resulting SNPs were analyzed using ChromoPainter (Lawson et al. 2012), assuming a uniform recombination map and running 10 iterations, maximizing over the recombination scaling constant. Table 2 lists the genomes included in this analysis.

### Identification and Annotation of *cps* Loci

**Fig. 2.—:**
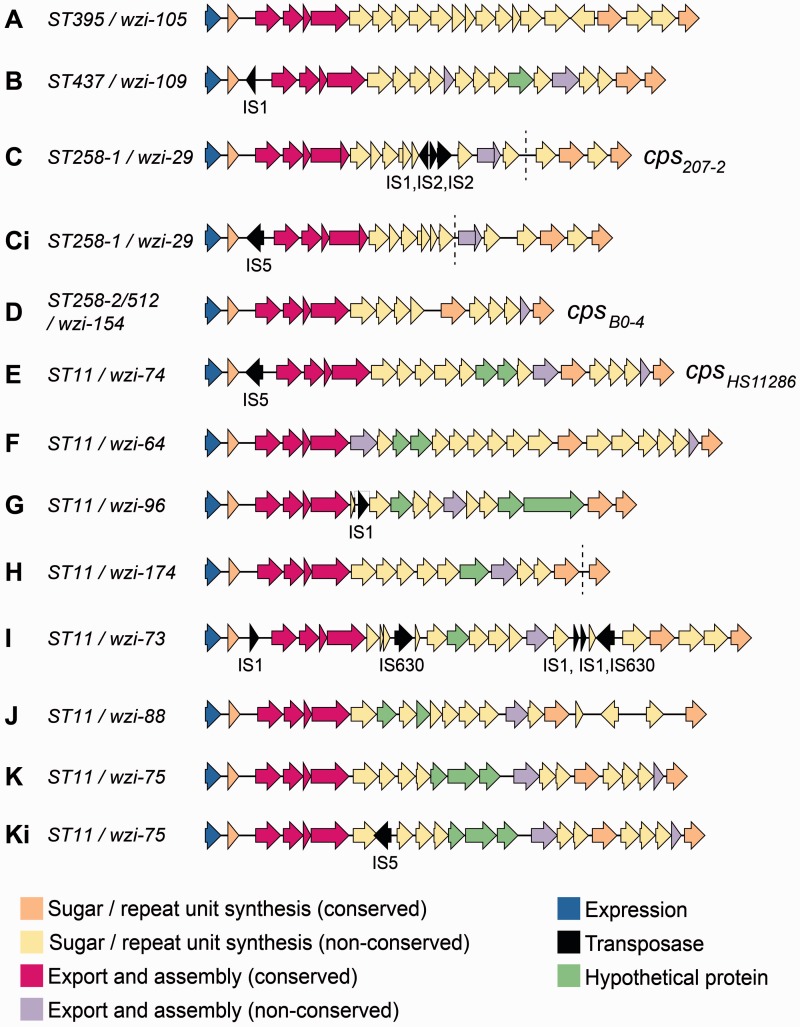
Structures of *cps* loci identified in *Klebsiella pneumoniae* CG258. Arrows indicate the direction, relative length, and function (colored as per legend) of protein-coding genes. Transposases were identified and labeled using the IS finder database (https://www-is.biotoul.fr/, last accessed April 23, 2015). *Cps* loci are labeled A through K as referred to in the text (table 1 and fig. 2); STs and *wzi* alleles are indicated; loci previously named in D’Andrea et al. (2014) are indicated (*cps_B0-4_*, *cps_207-2_*, and *cps_HS11286_*). *Cps* loci that vary from another only in the content and/or position of transposases are indicated by “i.” Dashed lines indicate contig breaks in assemblies of three loci.

For publicly available assemblies, genome sequences were retrieved from GenBank (accessions in table 1). For short read data, reads were assembled de novo using two alternative approaches: SPAdes (Bankevich et al. 2012; with kmers 21, 33, 55, 63, 71) and Velvet with Velvet Optimizer (Zerbino and Birney 2008). For each isolate, the assembly yielding the smallest number of contigs was used for the analysis (SPAdes assembly for all except DM23092/04 and 09-370B). *Cps* loci were identified and extracted from the assemblies using a custom Python script, whereby BLAST was used to identify sequence regions with homology to the flanking genes *galF* and *ugd* (nucleotide BLAST followed by protein BLAST if no nucleotide-level matches were found). When *galF* and *ugd* were not found on the same contig, the nucleotide sequences from, and including, *galF* or *ugd* up to the ends of their respective contigs were extracted. In such cases, contig adjacency was manually confirmed by visual inspection of PE read mapping to the *galF* and *ugd* contigs (reads were mapped against extracted *cps* locus sequences using BWA [Li and Durbin 2009], sorted and compressed with SAMTools [Li et al. 2009], and viewed in Artemis [Rutherford et al. 2000]). In three cases, the *galF* and *ugd* contigs could not be joined (i.e., *cps* loci were split across more than two assembly contigs) and additional contigs were identified by BLAST search of published *cps* locus sequences, and confirmed by read mapping. In three cases (VA360, KpMDU1, and 09-370B), a putative contig join could not be confirmed using the mapping approach. In the case of the public genome, ATCC BAA-2146, no reads were available for mapping. The annotated *cps* sequences for these loci therefore contain contig breaks, the positions of which are shown in figure 2.

*Cps* loci were clustered into groups representing distinct structures by visual comparison using BLAST and Artemis Comparison Tool (Carver et al. 2005). Single representatives of each distinct *cps* locus cluster were annotated using Prokka (Seemann 2014) together with a reference protein set derived from published *cps* loci available in GenBank (accessions: AB371289.1, AB198423.1, AB289646.1, AB289648.1, AB289650.1, AB290716.1, AB371296.1). The resulting annotations, available at https://github.com/kelwyres/Kp-cps-loci.git (last accessed April 23, 2015) and GenBank (accessions: KR007671–KR007677), were then manually inspected and curated. Nucleotide sequences of conserved coding sequences (CDSs) were extracted and pairwise similarities were calculated using MEGA5 (Tamura et al. 2011). Transposases were annotated using insertion sequence (IS) Finder (https://www-is.biotoul.fr/, last accessed April 23, 2015). *Wzi* alleles were assigned by comparison with the international *K. pneumoniae* BIGSdb (at http://bigsdb.web.pasteur.fr, last accessed April 23, 2015) using SRST2 (http://katholt.github.io/srst2/, last accessed April 23, 2015; dx.doi.org/10.1101/006627)*.*

### Pairwise Nucleotide Differences and Gene Distance Jaccard Scores

Nucleotide differences were calculated using SNP data generated as described above. SNPs representing putative recombinant genomic regions identified by BRATNextGen were excluded. The total set of genes represented among the CG258 genomes was identified by mapping to a pan genome sequence for CG258. The latter was obtained by using iterative contig comparison to collate a nonredundant set of distinct contig sequences (<95% sequence identity) present in the set of assemblies, which was annotated using Prokka (Seemann 2014). The presence of each gene in each read set was determined from mapping data, with presence defined as coverage of ≥95% of the length of the gene with mean read depth ≥5. Jaccard distances (*J*) were calculated as *J* = *a/b*, where *a* denotes the number of genes that were different between two genomes (i.e., present in one but not both) and *b* denotes the total number of genes present in either genome (i.e., present in one or both genomes).

## Results

### Definition of the Wider Clonal Group 258

It is generally accepted that *K. pneumoniae* of ST258 and its single locus variants, ST11, ST437, and ST512, represent a single clonal group, which descended from a recent common ancestor (Pereira et al. 2013; Deleo et al. 2014). We sought to identify all available genome sequences that may belong to CG258; therefore for the purposes of this analysis, we included all draft genomes of these STs that were available in public databases at the time of investigation (table 1). Additionally, MLST data and a core genome SNP phylogeny of a diverse global collection of *K. pneumoniae* (described elsewhere, supplementary fig. S1, Supplementary Material online) indicated that ST395 was related to ST11: ST395 shared 4/7 MLST loci with ST11, and we estimated 0.2% genome-wide divergence between ST11 and ST395 compared with 0.6% mean divergence between ST11 and other *K. pneumoniae* KpI. We therefore included the publicly available ST395 genome 1191100241, and our own sequenced ST395 isolate K242An, in the subsequent analysis.

### Evolution by Recombination

We used BRATNextGen (Marttinen et al. 2008) to assess the extent of recombination within the CG258 genomes (fig. 1). In total, 160 recombinant genomic regions were identified across the chromosome; these recombinogenic regions had a median length of 2,385 bp (range 21 bp to 1.47 Mb, mean 57 kb). The BRATNextGen analysis identified three large recombinant regions in the ST395 genomes, each of which was >100 kb. The largest of these regions (which span the origin in fig. 1) was approximately 1.5 Mb in length and contained the three MLST loci that differ from ST11 (*infB*, *mdh,* and *pgi*; fig. 1). One of the other two recombinant regions identified in ST395 (>500 kb in length) was shared in whole or in part by the ST11 and ST437 genomes (marked by * in fig. 1). This region spans the recently reported recombination into the most recent common ancestor (MRCA) of ST258 (Chen et al. 2014); we concluded that BRATNextGen incorrectly characterized this as an import into all strains except ST258, as opposed to an import into the MRCA of ST258, simply due to a lack of resolution to differentiate these two possibilities.

We sought to investigate and characterize the largest putative recombination events in more detail across these isolates. We screened the collection of 230 *K. pneumoniae* genomes for genetic markers (*wzi* and/or MLST alleles) matching those within the putative imported regions. We were unable to identify any putative donors of the large ST395 recombinant regions described above, but identified candidate donors for the ST258 lineage recombination events (ST42/*wzi-29* isolate DB44834/96 and ST442/*wzi-154* isolate QMP Z4-702).

We used ChromoPainter (Lawson et al. 2012) to estimate the probable ancestral origin of sites across the ST258-2 and ST258-1 genomes, as a function of our ST11, ST42, and ST442 genome sequences (supplementary fig. S2, Supplementary Material online). This analysis indicated that ST258-2 resulted from import into ST11 of a large ST442-like sequence spanning the *cps* locus and resulting in a change of *cps* type, followed by a later import of an ST42-like 50 kb sequence again spanning the *cps* locus, resulting in a further capsule switch and generating the ST258-1 lineage.

### Fine-Scale Phylogenetic Structure

A phylogeny reflecting vertical patterns of inheritance among the wider CG258 is shown in figure 1 (also see supplementary fig. S3, Supplementary Material online, ML tree inferred from genome-wide SNP calls after excluding SNPs introduced by putative recombination events identified by BRATNextGen). The MRCA for all ST11 genomes was close to the root of the tree, that is, the MRCA of the whole complex. Genomes of ST258 and ST512 formed a subcluster, within which two further sublineages were identified (supplementary fig. S3, Supplementary Material online), which matched those previously described as ST258-1, and ST258-2 plus ST512 (Deleo et al. 2014; Wright et al. 2014). The definition of these sublineages was supported even after putative recombinant genomic regions were removed from the analysis, as presented here and by Deleo et al. ST258/512 and ST395 were each defined as tight clusters separated by deep branches, indicating long-term divergence from the MRCA followed by recent independent clonal expansions of each sublineage. In contrast, the ST11 genomes exhibited a greater degree of diversity (fig. 1, panel B). Taken together, these phylogenetic and recombination analyses indicate that members of CG258 descend from a common ancestor (most likely of ST11) that has diversified into several distinct lineages, some of which have novel ST combinations due to point mutations or recombination affecting the MLST loci.

In all cases where dates were known, our *K. pneumoniae* were isolated between 2004 and 2012, and did not show any obvious patterns across the CG258 subclusters. All of the ST11 isolates originated from South East Asia, whereas the ST258/512 subcluster isolates originated from a wider geographic distribution including the United States, Italy, Israel, and Australia. However, given that our genomes do not represent a systematic sample, it is not possible to use our data to make any inferences about differences in geographic distribution between subclusters.

### *Cps* loci in CG258

We identified 11 distinct *cps* loci within CG258 (fig. 2, table 1). Three of these *cps* loci matched those previously characterized in the ST11 reference genome HS11286 (genotype *wzi-74*/*cps*_HS11286_, serotype K74) and in ST258/512 *K. pneumoniae* (genotypes *wzi-29*/cps_207-2_ and *wzi-154*/*cps*_BO-4_; unknown serotypes) (Chen et al. 2014; D’Andrea et al. 2014; Deleo et al. 2014). The remaining *cps* loci were annotated and are available in GenBank (accessions: KR007671–KR007677) or at https://github.com/kelwyres/Kp-cps-loci.git (last accessed April 23, 2015). The capsular serotypes were predicted based on *wzi* alleles (table 1).

**Fig. 3.—:**
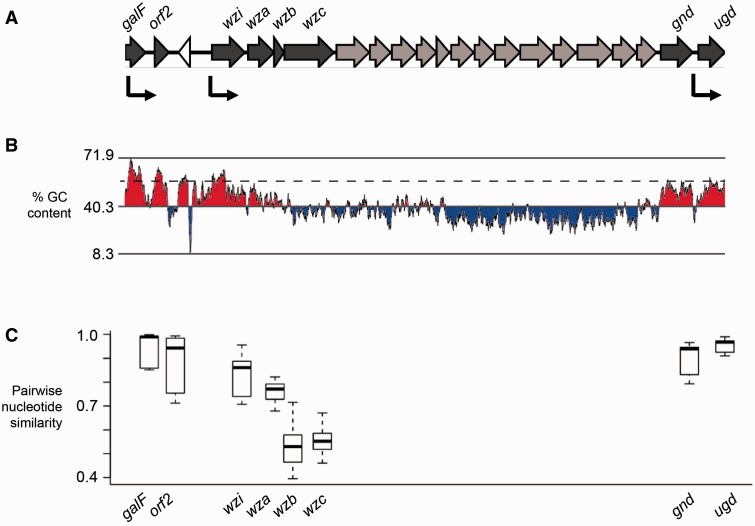
Macrostructure and features of *Klebsiella pneumoniae cps* loci. (*A*) Structure of a representative *cps* locus, *cps*-B (individual structures *cps* A–K are given in fig. 4). Filled arrows indicate size and direction of protein-coding genes; dark gray, conserved CDSs present in all *cps* loci (gene symbols labeled); light grey, nonconserved CDSs (present in *cps*-B but not other *cps* loci); white arrow, transposase. Black arrows indicate the relative start position and direction of transcriptional units as described in Shu et al. (2009). (*B*) G + C content across *cps*-B; red and blue indicate above and below the mean G + C content of the locus. Dashed line indicates mean G + C across the whole genome (57%). Note that although only *cps-B* is represented here, we observed a similar G + C content pattern across all *cps* loci. (*C*) Distributions of pairwise nucleotide similarities among CG258 *cps* loci, for conserved genes only.

All CG258 *cps* loci shared a conserved macrostructure consistent with that previously reported among *K. pneumoniae* (Shu et al. 2009) (figs. 2 and 3). The macrostructure comprised eight conserved protein CDSs situated at either end of the *cps* locus: *galF* (Uridine diphosphate-glucose pyrophosphorylase), *orf2* (putative acid phosphatase), *wzi*, *wza*, *wzb,* and *wzc* (polysaccharide polymerization and export) at the 5′ end, and *gnd* (6-phosphogluconate dehydrogenase) and *ugd* (UDP-glucose 6-dehydrogenase) at the 3′ end. The median pairwise nucleotide similarities within these genes ranged from 99% (*galF*) to 55% (*wzc*), with greatest genetic conservation observed at the terminal ends of the locus (fig. 3). The conserved CDSs located at either end of the locus had a Guanine + Cytosine (G + C) content of >50%, similar to the rest of the *K. pneumoniae* chromosome (the overall G + C content of the HS11286 chromosome was 57.5%). In contrast, the nonconserved CDSs in the center of the *cps* loci had <50% G + C content (fig. 3). These data suggest that the evolutionary origins of the nonconserved CDSs are distinct from those of the conserved CDSs and the rest of the *K. pneumoniae* chromosome. Presumably, the central CDSs have been transferred horizontally into the center of the locus and then exchanged through homologous recombination mediated by sequence conservation in the outer CDSs.

The *cps* sequences indicated that variation in the central region drives differences in the overall length and CDS content of the *cps* locus, which ranged from 20 to 31 kb and from 16 to 24 CDSs, respectively (excluding transposases, see fig. 2). Among the 223 independent CDSs annotated across the 11 CG258 *cps* loci, predicted protein products included sugar production/processing proteins (*n* = 69), sugar transferase proteins (*n* = 69), capsule export and assembly proteins (*n* = 44), sugar transport proteins (*n* = 15), unknown proteins associated with capsule production (*n* = 11), and hypothetical proteins (*n* = 15) (fig. 2). The nonconserved CDSs in the center of the locus were predominantly associated with capsule-specific sugar synthesis and assembly. For example, *cps*-A, -F, and -I carried *manB* and *manC* that encode a phosphomannomutase and a mannose-1-phosphate guanylyltransferase, respectively. In total, 134 CDSs were identified within the nonconserved *cps* regions. BLAST comparisons with nucleotide and protein sequences in the National Center for Biotechnology Information (NCBI) database indicated that 58 of these proteins were associated with the synthesis, processing, and/or export of specific sugars and/or sugar derivatives: Mannose (*n* = 21), galactose (*n* = 9), glucose (*n* = 9), colonic acid (*n* = 8), pyruvic acid (*n* = 5), acetic acid (*n* = 4), fucose (*n* = 1), and hyaluronic acid (*n* = 1). A further 61 CDSs had ≥70% amino acid identity to sequences commonly associated with sugar synthesis, processing, and/or export. Fifteen CDSs did not match any known nucleotide or protein sequences in the NCBI database or matched sequences annotated as hypothetical. Notably, the nonconserved region of the *cps* locus of National Institutes of Health (NIH) outbreak-associated isolates included two putative rhamnosyltransferase genes, consistent with the reported detection of rhamnose derivatives in the capsule of outbreak-associated isolates (Kubler-Kielb et al. 2013).

Thirteen transposase-associated CDSs were additionally identified within the *cps* sequences (fig. 2). These included IS1 (present in four *cps*), IS2 (present in one *cps*, adjacent to an IS1 insertion), IS5 (present in three *cps*), and IS630 (present in one *cps*). Furthermore, four of the IS insertions (two IS1, two IS5) occurred upstream of *wzi*, near the transcriptional start site for the majority of capsular synthesis genes. These transposases have strong promoters and are in frame with the *cps* CDS. The other IS insertions all occur within the central sugar processing regions—two of these disrupt CDS (IS1 in *cps*-G and IS5 in *cps*-Ki).

For two *cps* loci, transposase insertions were differentially present, generating variant forms of the *cps* locus. *Cps*-K (*wzi*-75), identified in three closely related ST11 isolates from Singapore, carried an IS5 insertion in the sugar-processing region in one isolate. Both available ST258-1 references carried a copy of the *cps*-C (*cps*_207-2_) locus with either an IS5 insertion upstream of *wzi* or an IS1 and two IS2 insertions within the central sugar-processing region. The *cps*-C locus in the donor strain ST42 and all ST258-1 strains reported in Deleo et al. (2014) carried the IS1/IS2 insertion without the IS5 insertion. It is likely that the *cps*-C sequence imported into ST258 from ST42 was in the form of *cps*-C (containing the IS1/IS2 insertion), but has since diversified though loss of the IS1/IS2 insertions and acquisition of the IS5 insertion in some strains (*cps*-Ci).

### Phylogenetic Distribution of *cps* Loci and Capsule Switching

**Fig. 4.—:**
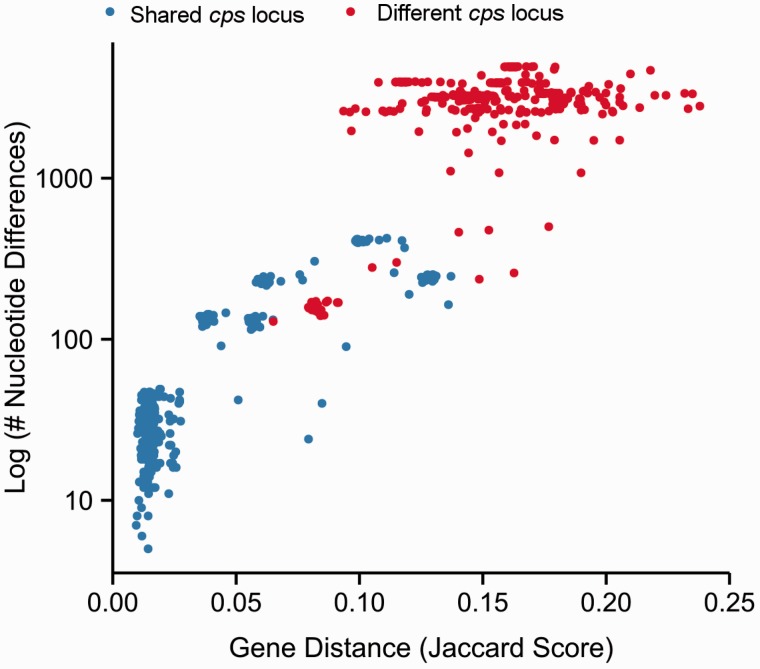
Total nucleotide differences versus gene content distances (Jaccard scores) for pairs of CG258 *Klebsiella pneumoniae.* Points are colored blue for genome pairs that share a *cps* locus structure, red otherwise.

The various *cps* loci were confined to distinct phylogenetic subclusters within CG258 (fig. 1). Across the clonal group, genomes with different *cps* regions were also differentiated in terms of nucleotide divergence across conserved regions of the genome (fig. 4). As presented above, the ST258/512 cluster harbored two *cps* loci, each confined to one of the ST258 sublineages that were separated by a few hundred SNPs. The next closest genome pair with different *cps* loci was NCSR101 and ATCC BAA-2146 (both ST11), which differed by 500 SNPs across the rest of the genome, whereas all other pairs of genomes with different *cps* loci differed by >1,000 SNPs. These data suggest that stable capsule switching events may occur as frequently as one in every 10^2^–10^3^ nucleotide substitutions in *K. pneumoniae.* (A recent molecular clock analysis of ST258 *K. pneumoniae* suggested that this may be equivalent to as few as 3 years in real time; Gaiarsa et al. (2015). Extensive differences in gene content were also observed within CG258 and were correlated with nucleotide divergence (fig. 4). Genomes with different *cps* loci also differed substantially in terms of gene content outside the *cps* locus (mean 809 genes different between pairs of genomes).

## Discussion

Our data provide important insights into the evolution and capsular diversity of globally distributed KPC-associated *K. pneumoniae* CG258. Significantly, we show that the previously reported large-scale recombination events and capsule switches are not unique events in the history of this clonal group, but are a major and potentially common driver of variation within CG258. Our analyses also provide independent evidence to support the previous findings that ST258/512 emerged as a hybrid lineage within CG258 (Chen et al. 2014; Wright et al. 2014) and, through use of an alternative analytical framework (ChromoPainter) and novel genomes, we provide independent confirmation of the large-scale recombination events that drove its emergence and resulted in capsule switching.

**Fig. 5.—:**
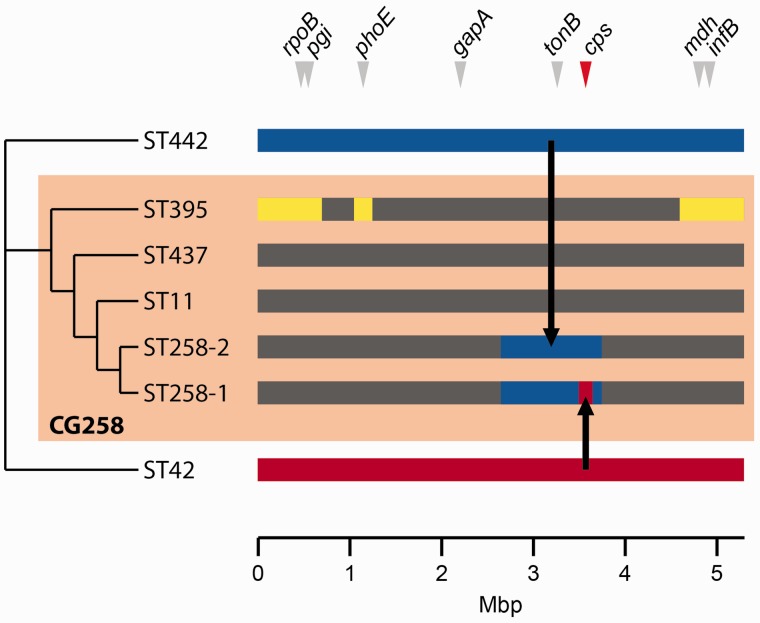
Evolutionary history of the CG258 genomes. Dendrogram represents hierarchical relationships among ST442, ST42, and the CG258 STs (branch lengths not meaningful). Colored bars (right) represent the genome of each ST (approximate scale only, coordinates relative to HS11286 genome). Dark grey blocks represent genomic regions descended from the MRCA of CG258; yellow blocks represent recombinant regions acquired from an unknown donor(s); dark blue bar represents the ST442 genome, part of which was imported to ST11 forming the hybrid ST258-2 genome; red bar represents the ST42 genome, part of which was imported to ST258-2 forming ST258-1. The positions of MLST loci and the *cps* locus are indicated by arrows.

We identified a large (1.5 Mb) recombination import and a second import of approximately 113 kb within the ST395 genomes (fig. 5), which lay within CG258 (fig. 1). These large-scale recombination events led to the acquisition of divergent alleles at three of the seven *K. pneumoniae* MLST loci (fig. 5). As a consequence, ST395 (a 4-locus variant of ST258) has not routinely been considered part of the global CG258, even when KPC ST395 was found cocirculating with KPC ST11 in Asia (Yang et al. 2013) or when CGs were defined based on core genome MLST (MLST comprising 694 conserved *K. pneumoniae* genes, whereby CGs were defined as groups of isolates that differed at ≤100 loci) (Bialek-Davenet et al. 2014). Our data indicate that ST395 is highly similar to ST258 in that it is a hybrid KPC-associated strain emerging within CG258. Our analysis therefore provides strong evidence for an expanded definition of CG258 to include all strains that share an MRCA with ST11 as opposed to being based on shared MLST alleles (fig. 1). Furthermore, our data suggest that all CG258 isolates, including ST395 and ST437, should be included in any surveillance and research investigations focused on KPC CG258.

We identified 11 distinct *cps* loci among CG258, of which only 3 had been previously described in the complex (D’Andrea et al. 2014). In addition, further IS-associated variants of two loci were identified (fig. 2). The 11 *cps* loci were differentiated by structural (gene content and synteny) rather than nucleotide-level differences. As such, the presence of multiple different variants among otherwise closely related isolates indicates change by horizontal transfer rather than mutation. In contrast to the large recombination events identified in ST395 and ST258, most *cps* locus changes were not associated with large-scale recombination events, suggesting that the recombination breakpoints associated with these capsule switches lay much closer to, or possibly within, the *cps* locus itself. This hypothesis is consistent with the observation of declining sequence conservation toward the center of the *cps* locus, because this region has likely been affected by a greater number of recombination events than the outer *cps* locus regions throughout the evolutionary history of *K. pneumoniae.* Alternatively, it is possible that the downstream recombination breakpoints lay outside of the *cps* locus but were masked by interstrain variation at the nearby Lipopolysaccharide locus (O antigen locus), which also varied among our *K. pneumoniae* genomes. The latter may be indicative of diversification of LPS within the complex; however, as the genetics of LPS production in *K. pneumoniae* are not well-understood, we are unable to draw conclusions about this from the sequence data.

The capsular serotypes of the ST258 isolates included in this study were unknown, as serotyping is rarely performed for *K. pneumoniae.* However, previous studies have reported expression of serotype K41 among three ST258 *K. pneumoniae* that were tested (Tzouvelekis et al. 2013). It is not clear which of the ST258-associated *cps* loci was present in those serotyped isolates, although it was noted that they harbored KPC allele 2, which has been associated with ST258-1/*wzi-29*/cps_207-2_ (Deleo et al. 2014; Wright et al. 2014). The *cps* loci reported here had very few conserved genes (figs. 2 and 3) and differed extensively in their complement of sugar-processing genes. Hence, while the associated serotypes are not known for all the *cps* loci, exchange of these loci within CG258 can be assumed to result in phenotypic capsule switching.

The *cps* loci were correlated with phylogenetically defined sublineages (fig. 1). We also observed diversification of *cps* loci into variants within phylogenetic sublineages, via the activity of IS, which may prove to be useful epidemiological markers for identifying ST258 subclones. The ISs involved are diverse and are not site specific, but were nevertheless observed only in two regions within the *cps*—upstream of *wzi*, or within the central sugar-processing region. Only 2 of the 13 insertions interrupted coding sequences, indicative of selection against transposition events that interrupt expression of the capsular biosynthesis genes (as other IS insertions within CDSs presumably occur but are deleterious in competition with encapsulated strains). However, the IS insertions could hypothetically alter capsular gene expression, or promote capsule switching via rearrangement or horizontal transfer. IS1 and IS5 were identified upstream of *wzi*, within the promoter region of the majority of capsular biosynthesis genes (Shu et al. 2009), in four different *cps* loci (figs. 2 and 3). It has been shown that insertion of IS5 into specific sites upstream of coding sequence regions can enhance expression of various operons in *E. coli* (Schnetz and Rak 1992). IS1 insertions in either orientation can also enhance (Olliver et al. 2005) or interrupt (Fernández et al. 2007) gene expression in *E. coli.* Furthermore, insertion of IS1301 upstream of the capsule biosynthesis (*sia*) and export (*ctr*) operons of *Neisseria meningitidis* C has been shown to upregulate capsule expression and promote resistance to complement-mediated killing (Uria et al. 2008). Therefore, the apparent hotspot for IS acquisition upstream of *wzi* in the *K. pneumoniae cps* locus may indicate not only purifying selection against deleterious mutants resulting in loss of capsular expression, but also positive selection for enhanced capsule expression at the transcriptional level. In addition, IS1, IS2, IS5, and IS630 were all found within the sugar-processing region of the *cps* loci. IS1-mediated rearrangement of the *cps* locus has been reported in *E. coli* (Drummelsmith et al. 1997); consequently, the accumulation of IS in this central region may contribute to capsule switching and diversification of the *cps* loci in *K. pneumoniae.*

Extensive capsular switching within clones is well-understood in Gram-positive pathogens such as *Streptococcus pneumoniae*, and was recently reported among epidemic *Acinetobacter baumannii* (Kenyon et al. 2013) and *E. coli* (Alqasim et al. 2014), but has not been widely reported among other Gram-negative *Enterobacteriaceae* (Croucher and Klugman 2014). The high number of distinct *cps* locus variants within our sample suggests that capsule switching may be a common event across the wider *K. pneumoniae* CG258. It is reasonable to assume that as the ST258 sublineages continue to evolve, their *cps* loci will continue to diversify through recombination, transposition, and potentially transposase-mediated horizontal gene transfer, as described here for ST11 and other members of the wider CG. The pool of potential *cps* locus donors is expansive; 78 capsular serotypes have been defined, and *wzi* and *wzc* sequencing efforts suggest that many more *cps* loci exist in the wider *K. pneumoniae* population (Brisse et al. 2013; Pan et al. 2013). The ST442/*wzi-154* and ST42/*wzi-29K. pneumoniae* we identified as being closely related to the donors involved in ST258 recombination events were isolated under very different circumstances to those described in previous reports. The *K. pneumoniae* ST442/*wzi-*154 strain in this study was isolated from a case of bovine mastitis at a dairy farm in New York State in 2006 (Kanevsky-Mullarky et al. 2014), which is geographically close to the location of the first reports of ST258 in New York in 2000 (Woodford et al. 2004). The ST42/*wzi-*29 was isolated in 1996 from a blood stream infection in a hospital in Singapore (Koh et al. 1999) (note that ST11 is considered most common in South East Asia). Thus, our data serve as a reminder that circulating genetic variation, including virulence determinants such as the *cps* locus, can be disseminated through both clinical and environmental sources. The latter niche is currently drastically undersampled, meaning that much of the genetic variation circulating within global *K. pneumoniae* populations is not yet captured.

It is not yet clear whether capsular switching is equally common among all *K. pneumoniae* CGs. Evidence from our data suggest that a similar pattern of capsular diversity may exist among ST42 *K. pneumoniae* (we identified four genomes with three different *cps* loci: One with *wzi*-29, one with *wzi*-41, and two with *wzi*-33—all unknown serotypes; genome data in BIGSdb, http://bigsdb.web.pasteur.fr, last accessed April 23, 2015). In contrast, ST23 *K. pneumoniae* have generally been associated with a single capsule type (Bialek-Davenet et al. 2014). In order to better understand the capsule epidemiology among different *K. pneumoniae* CGs, it may be valuable to include capsule typing methods in routine surveillance programs, perhaps utilizing *wzi* or *wzc* sequencing as an indication of *cps* locus diversity (although it should be noted that the extent to which *wzi/wzc* alleles are conserved among structurally similar *cps* loci encoding the same capsule synthesis machinery, and vice versa, is not yet known). However, these data should not be used to inform epidemiological investigations in the absence of core chromosomal information such as MLST or whole genome SNPs, because distantly related *K. pneumoniae* may harbor highly similar *cps* loci, for example, the ST258/*wzi-29* and ST42/*wzi-29* isolates described here.

Given that *K. pneumoniae* capsule variants are immunologically distinct (Campbell et al. 1996), the apparent propensity of KPC CG258 to undergo multiple sequential capsular switching is concerning, and suggests that capsular-based vaccines or depolymerase treatments may be of limited use. One possible vaccination strategy would be to target a wider range of capsule types, similar to the approach currently used in the design of *S. pneumoniae* capsular vaccines (currently 10 or 13 types). However, comprehensive population surveillance would be required in order to monitor the response to vaccination, which in the case of *S. pneumoniae* has included the emergence of vaccine escape strains (Golubchik et al. 2012) and the expansion of pre-existing clones expressing capsule types not targeted by the vaccine (Pai et al. 2005). Depolymerase treatment comprises the application of capsule-specific phage enzymes that lyse *K. pneumoniae* capsules. Such treatment has been shown to improve the survival rate of mice infected intraperitoneally with *K. pneumoniae* (Lin et al. 2014), but would likely be difficult to scale in the face of extensive capsule diversity. In either case, a more comprehensive understanding, including investigation of diversity and evolution of capsular loci among the broader population of clinical, human carriage and environmental *K. pneumoniae* isolates, is required.

## Supplementary Material

Supplementary figures S1–S3 are available at *Genome Biology and Evolution* online (http://www.gbe.oxfordjournals.org/).

Supplementary Data
