# Supplementary material for: Extensive Capsule Locus Variation and Large-Scale Genomic Recombination within the Klebsiella pneumoniae Clonal Group 258
Source: Genome Biol Evol. 2015 Apr 10;7(5):1267–79. doi: 10.1093/gbe/evv062 (PMC4453057; doi:10.1093/gbe/evv062)
Supplement: Supplementary Data [file supp_7_5_1267__index.html]

Extensive capsule locus variation and large-scale genomic recombination within the Klebsiella pneumoniae clonal group 258 — Extensive Capsule Locus Variation and Large-Scale Genomic Recombination within the Klebsiella pneumoniae Clonal Group 258 — Supplementary Data 

# Extensive Capsule Locus Variation and Large-Scale Genomic Recombination within the *Klebsiella pneumoniae* Clonal Group 258

## Supplementary Data

files

**Files in this Data Supplement:**

- Supplementary Data - doc file
