# Supplementary material for: Extensive Capsule Locus Variation and Large-Scale Genomic Recombination within the Klebsiella pneumoniae Clonal Group 258
Source: Genome Biol Evol. 2015 Apr 10;7(5):1267–79. doi: 10.1093/gbe/evv062 (PMC4453057; doi:10.1093/gbe/evv062)
Supplement: Supplementary Data [file supp_evv062_Wyres_GBE_Supplementary.doc]

**Supplementary Material for: Extensive capsule locus variation and large-scale genomic recombination within the *Klebsiella pneumoniae* clonal group 258**

Kelly L. Wyres^1,2^*, Claire Gorrie^2^, David J. Edwards^2^, Heiman FL Wertheim^3^, Li Yang Hsu^4^, Nguyen Van Kinh^3^, Ruth Zadoks^5^, Stephen Baker^6^, Kathryn E. Holt^2^

^1^ IBM Research—Australia, Level 5, 204 Lygon Street, Carlton, Victoria 3053, Australia.

^2^ Bio21 Institute, University of Melbourne, 30 Flemington Road, Parkville, Victoria 3052, Australia.

^3^ Wellcome Trust Major Overseas Programme, Oxford University Clinical Research Unit, Hanoi, Vietnam; Nuffield Department of Clinical Medicine, University of Oxford, Oxford, OX3 7BN, UK.

^4^ National Hospital for Tropical Diseases, Hanoi, Vietnam.

^5^ Institute of Biodiversity, Animal Health and Comparative Medicine, College of Medical, Veterinary and Life Sciences, University of Glasgow, Garscube Campus, Glasgow G61 1QH, UK; Moredun Research Institute, Pentlands Science Park, Bush Loan, Penicuik, Midlothian EH26 0PZ, UK.

^6^ Wellcome Trust Major Overseas Programme, Oxford University Clinical Research Unit, Ho Chi Minh City, Vietnam; Centre for Tropical Medicine, Nuffield Department of Clinical Medicine, Oxford University, Oxford, OX3 7BN, UK; The London School of Hygiene and Tropical Medicine, London, WC1E 7HT, UK.


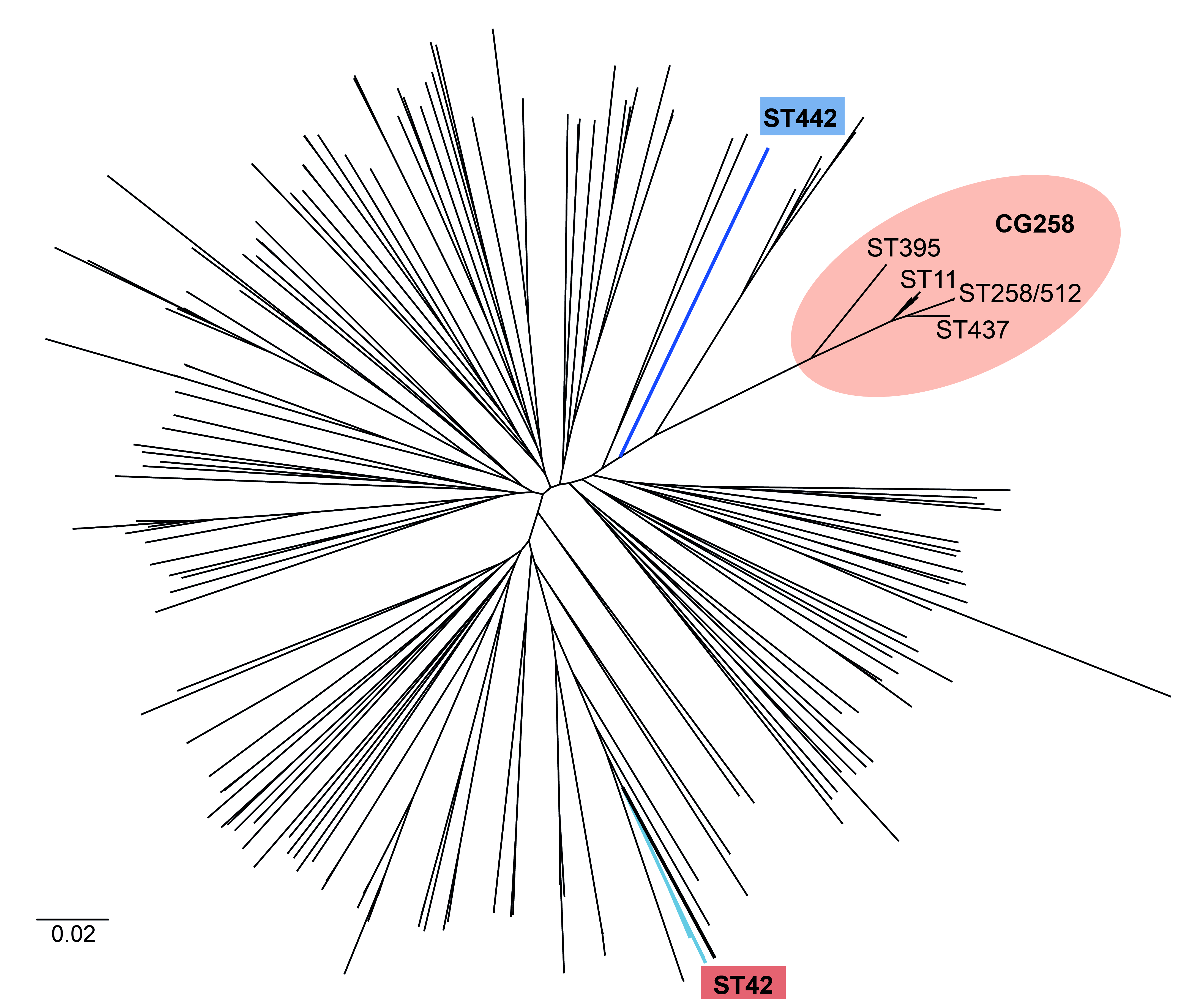


**Supplementary Fig S1. Whole-genome SNP phylogeny for 261 *K. pneumoniae* KpI genomes.**

Two-hundred and thirty genomes were from our global collection and 31 genomes were publicly available (see Methods). CG258 is highlighted, with sequence types (STs) marked. Genomes representing ST442 and ST42 are indicated. ST442 and ST42 are the putative donors of the large-scale genomic imports resulting in the evolution of ST258-2 and ST258-1, respectively.


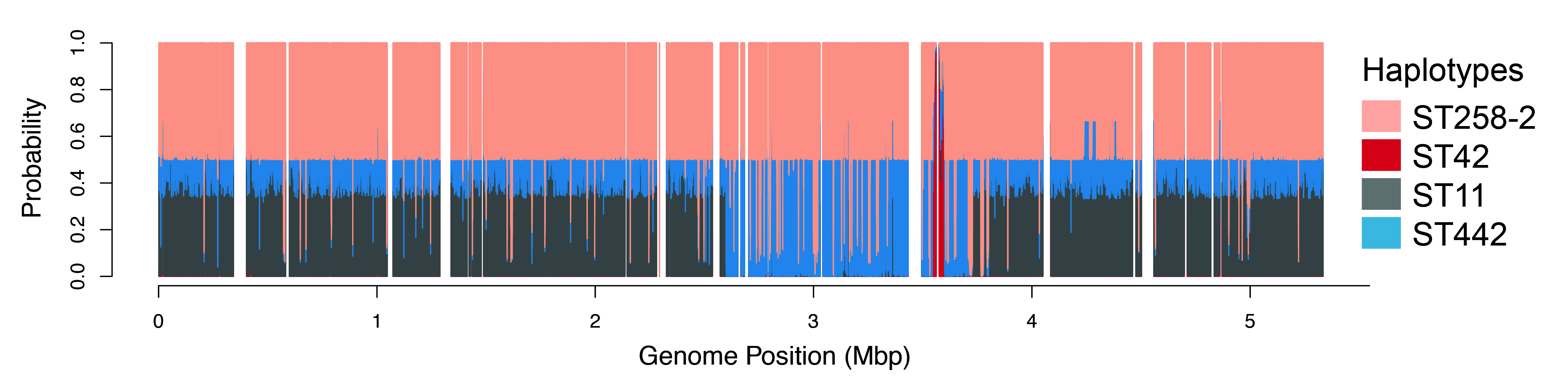


**Supplementary Fig S2. Probable ancestral origins of the ST258-1 genome.**

Ancestral origins were inferred using ChromoPainter to express the probability of each position in the genome deriving from each of ST258-2, ST11, ST42 and ST442. Most of the ST258-1 genome shares equal similarity (~0.5 each) with ST11 (grey) and ST258-2 (peach), with the exception of a large central segment that is derived from ST442 (dark blue) rather than ST11, and a small section spanning the *cps* (capsular synthesis) locus that differs between the two ST258 variants and is derived from ST42 (red). Genome coordinates as per NJST258_1 (ST258-1) reference. Note, non-variable genome positions are not shown.


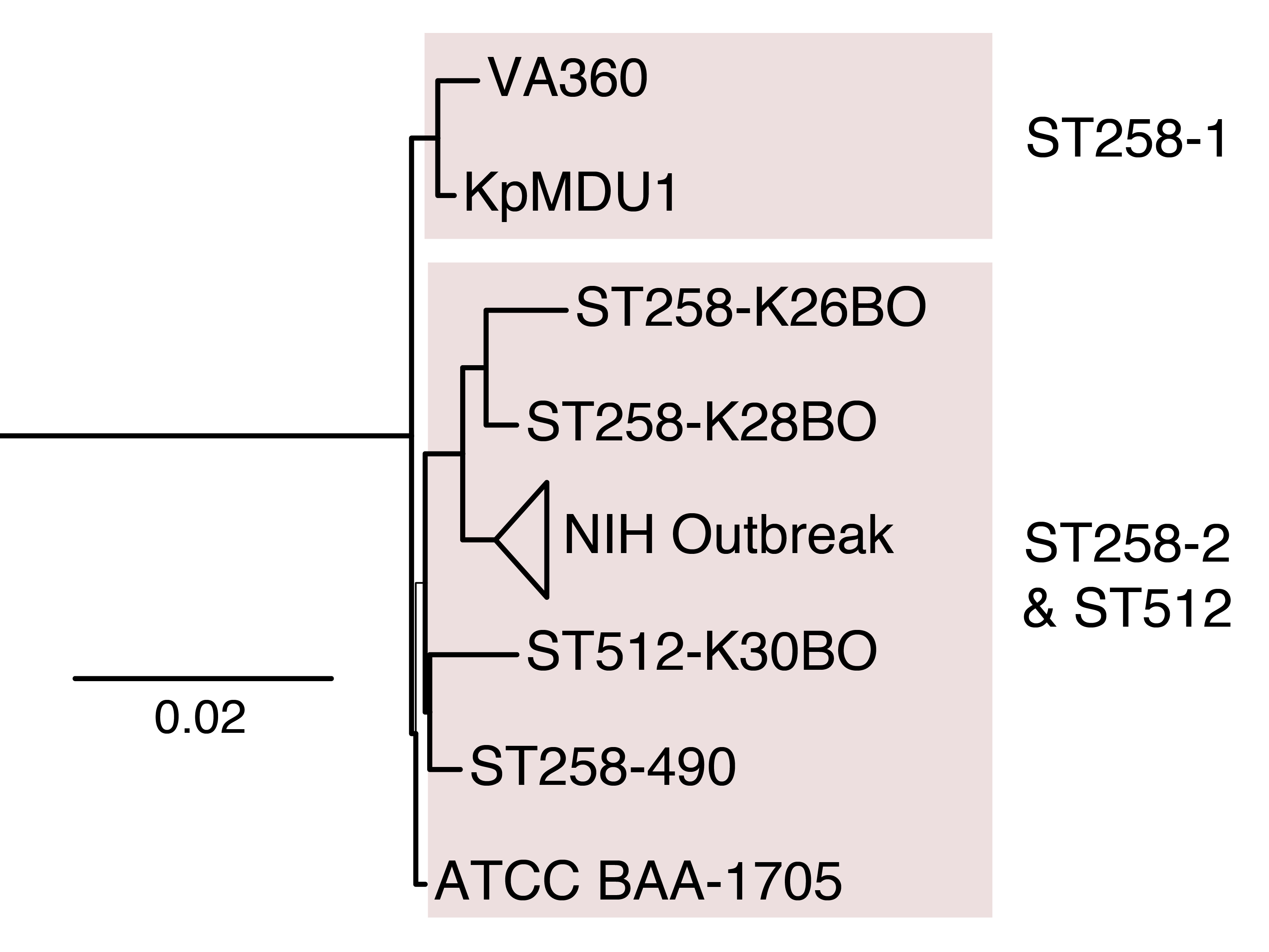


**Supplementary Fig S3. High resolution view of the phylogenetic relationships among ST258/512 *K. pneumoniae.***

The phylogeny represents a subsection of that shown in Figure 1, generated using genome-wide polymorphisms, excluding recombinant regions identified by BRATNextGen. The ST258-1 and ST258-2/512 sublineages are marked.
